# The interactions of spontaneous abortion, dietary intake of selenium, and fat mass and obesity associated (FTO) genotype: a case-control study in Iran

**DOI:** 10.3389/fnut.2024.1428648

**Published:** 2024-12-20

**Authors:** Narjes Nooriani, Zahra Saeedirad, Soheila Shekari, Sheyda Nami, Zahra Mahmoudi, Khadijeh Abbasi Mobarakeh, Somayyeh Bararnia Adabi, Shiva Khodarahmi, Parsa Bahmani, Saeid Doaei, Marjan Ajami, Maryam Gholamalizadeh

**Affiliations:** ^1^Department of Community Nutrition, School of Nutrition and Food Sciences, Isfahan University of Medical Sciences, Isfahan, Iran; ^2^Department of Clinical Nutrition and Dietetics, Faculty of Nutrition and Food Technology, Shahid Beheshti University of Medical Sciences, Tehran, Iran; ^3^Department of Nutrition, Science and Research Branch, Islamic Azad University, Tehran, Iran; ^4^Department of Clinical Biochemistry, Iran University of Medical Sciences, Tehran, Iran; ^5^Department of Community Nutrition, Nutrition and Food Security Research Center, School of Nutrition and Food Science, Isfahan University of Medical Sciences, Isfahan, Iran; ^6^Student Research Committee, Department of Nutrition, Faculty of Medicine, Urmia University of Medical Sciences, Urmia, Iran; ^7^School of Nursing and Midwifery, Shahid Beheshti University of Medical Sciences, Tehran, Iran; ^8^School of Nursing and Midwifery, Hamadan University of Medical Sciences, Hamadan, Iran; ^9^Department of Community Nutrition, Faculty of Nutrition Sciences and Food Technology, National Nutrition and Food Technology Research Institute, Shahid Beheshti University of Medical Sciences, Tehran, Iran; ^10^Reproductive Health Research Center, Department of Obstetrics and Gynecology, School of Medicine, Al-Zahra Hospital, Guilan University of Medical Sciences, Rasht, Iran; ^11^Department of Food and Nutrition Policy and Planning, School of Nutrition Sciences and Food Technology, National Nutrition and Food Technology Research Institute, Shahid Beheshti University of Medical Sciences, Tehran, Iran; ^12^Cancer Research Center, Shahid Beheshti University of Medical Sciences, Tehran, Iran

**Keywords:** spontaneous abortion, abortion, diet, selenium, FTO gene

## Abstract

**Background:**

Spontaneous abortion (SA) is reported to be associated with Fat Mass And Obesity-Associated FTO genotype and dietary intake of selenium. This research assessed the potential interactions between the risk of SA, dietary selenium intake, and the FTO rs9939609 polymorphism.

**Methods:**

This case-control study encompassed 192 women who experienced SA and 347 control participants. Dietary selenium intake was evaluated using a comprehensive food frequency questionnaire (FFQ) and Nutritionist IV software. The FTO gene was genotyped for rs9939609 polymorphism.

**Result:**

The findings showed that there were no significant variations in the case and control groups’ dietary selenium intake. A lower selenium intake was inversely associated with SA only among individuals with the TT genotype of the FTO gene (*β* = −0.19, *p* = 0.04). The results remained unchanged when age, BMI, physical activity, smoking, alcohol consumption, and calorie intake were taken into account.

**Conclusion:**

A link may exist between selenium consumption and SA, especially in individuals with the TT genotype in the FTO gene. These findings underline the influence of genetic factors on how dietary intake impacts SA. Further investigation is required to validate these conclusions.

## Introduction

Spontaneous abortion (SA), also known as miscarriage, can occur before a pregnancy has been discovered and can involve several complications like an empty gestational sac, halted embryonic growth, embryo or fetus demise, and embryo expulsion and its associated tissues. SA occurs before 20 weeks’ gestation depending on various sources and definitions (1–4) and reproductive-age women have a 10% likelihood of experiencing SA (5). These women are predisposed to experiencing bleeding and infection, considered the main contributors to maternal mortality. Parents, especially mothers, face immense difficulties including various psychological, emotional, physical, and cognitive/behavioral issues that should struggle with them (6).

Several factors have been found to have an impact on the probability of SA. These include lifestyle choices such as diet, maternal smoking, and alcohol consumption. Additionally, other factors like obesity, maternal age of 35 years or older, prior history of miscarriage, ectopic pregnancy, anemia, urinary tract infection, and high blood pressure during pregnancy have also been associated with an increased risk of SA (7–12). Several studies demonstrated a significant association between vital micronutrients such as selenium, zinc, and copper, and the risk of reproductive issues. It has been reported that selenium is crucial in maintaining the proper functioning of the reproductive system so insufficient selenium can cause gestational complications, miscarriages, and adverse effects on the fetus’s nervous and immune systems (13). According to a recent study, selenium is an essential micronutrient that plays a vital role in reproductive health and both deficiency and excess of selenium have been linked to reproductive problems like gonadal insufficiency and gamete dysfunction in both males and females (14). Such issues can lead to the failure of embryo implantation, distorted embryonic development, and eventually, sexual dysfunction and infertility (15). Also, selenium has been identified as a crucial component of reproductive enzymes, with increasing demand during pregnancy due to the fetus’s absorption of selenium (16).

On the other hand, genome-wide association studies found that the fat mass and obesity-associated gene (FTO) is involved in several metabolic diseases (17, 18). Abnormal methylation and oxidative stress resulting from decreased FTO expression in the chorionic villi affect immune tolerance and angiogenesis at the interface between the mother and fetus, ultimately leading to spontaneous abortion (19). Furthermore, some studies indicated that genetic variations in FTO could potentially impact weight gain and weight-related diseases by either increasing the amount of food consumed or affecting the regulation of appetite and satiety. The presence of FTO rs9939609 SNPs has been associated with increased intake of macronutrients, particularly fat and carbohydrates (20). However, research on the interaction of the FTO gene and selenium on the occurrence of SA remained limited. So, the purpose of this study was to investigate and evaluate the connection between FTO genotypes, selenium, and SA.

## Materials and methods

### Study population

This case-control study was initially conducted on 600 reproductive women, including 200 women with abortion and 400 women without a history of SA in Tehran, Iran. The sample size was calculated using OpenEPI online software (21) and the odds ratio obtained in a similar study (22) Participants were chosen at random women who were referred to Shohadaye Tajrish Hospital for routine medical examination. The inclusion criteria for the case group included a history of at least one placental loss before the 20th week of pregnancy, be recently diagnosed (within 3 months) and being between the ages of 20 and 40. The inclusion criteria for the control group were no history of SA and age between 20 and 40 years. Those who were taking selenium supplements (*n* = 7), did not wish to continue participating in the study (*n* = 21), or were unable to provide the necessary information (*n* = 33), were excluded from the study. Final analyses were performed on 539 participants including 192 women with abortion and 347 women without a history of SA. In the initial stages of the investigation, all participants were informed about the purpose and the method of the study. and a written consent was obtained from them as well.

### Data collection and measurements

A standard general questionnaire was used to collect data related to various factors such as age, education level, medical history of reproductive system diseases, diabetes and hypertension, history of abortion and pregnancy, smoking, and alcohol consumption through in-person interviews. A caliper tool with a 0.5 cm accuracy was used to measure the person’s height, and a digital scale (Seca, Germany) with a 0.5 kg accuracy was used to determine their weight.

### Genotyping of the FTO gene

**Figure 1:**
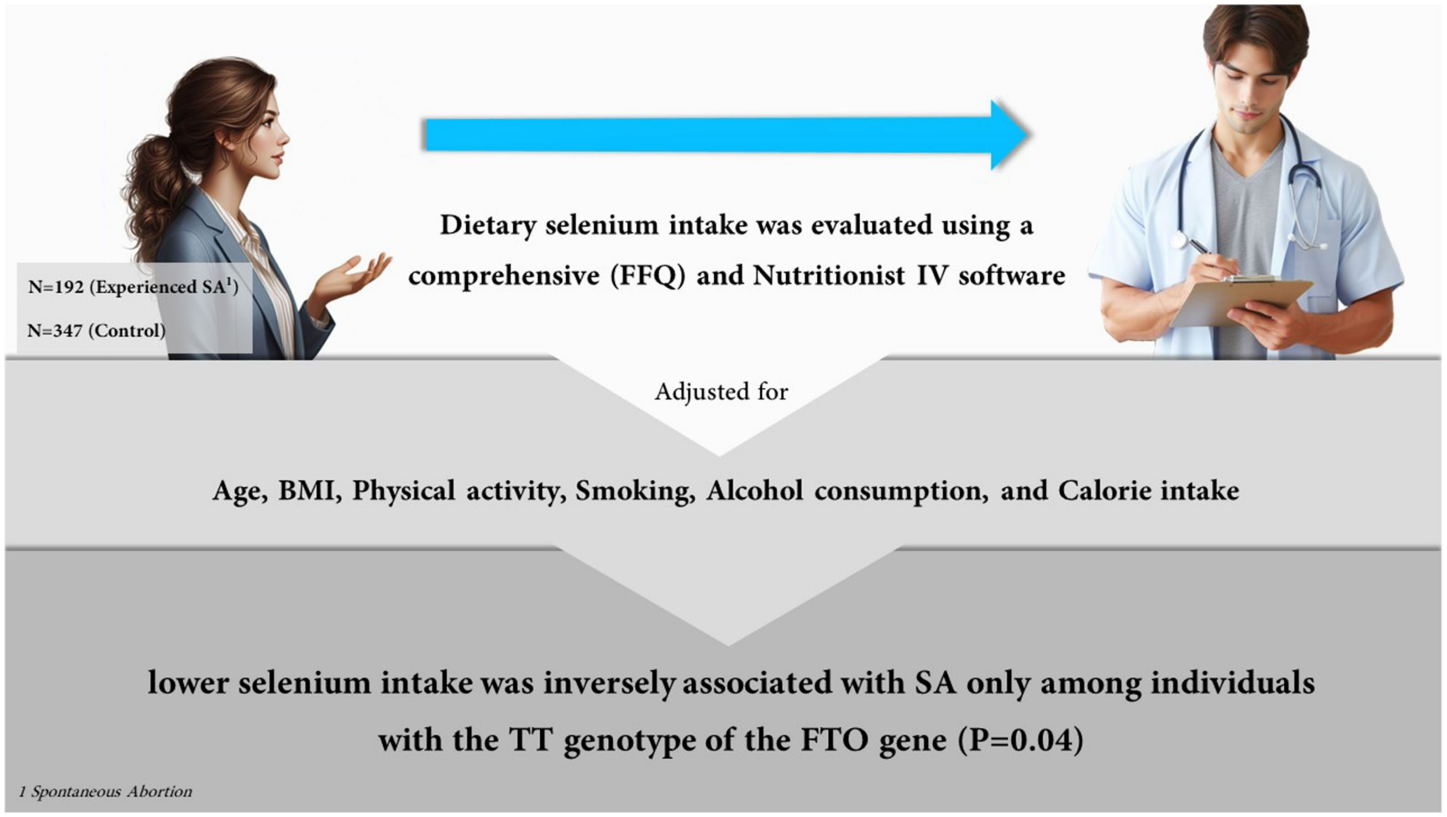
The assosiation between selenium, abortion, and FTO polymorphisms. Created by Copilot (2024), powered by OpenAI’s DALL-E 3 model, integrated alongside the GPT-4 language model.

For assessing the genotype of the FTO gene concerning rs9939609 polymorphism, 5 cc of blood was collected from all participants. Then, blood samples were centrifuged at 2,500 × g for 15 min and buffy coats were then separated. A standard kit (Gene All Company, South Korea) was used to extract DNA. The NanoDrop device (Thermo Scientific, Wilmington, DE, United States) was applied to quantify DNA concentration. The optical density (OD) of the samples was measured at a wavelength of 260–280 nm. The quality of the extracted DNA was assessed by agarose gel electrophoresis. About 200 ng DNA was taken for the amplification of the FTO gene using the PCR method and the DNA Polymerase Master Mix DNA Polymerase (cat. No. A180301; Ampliqon, Denmark) (23) (Figure 1).

### Dietary intake of selenium

Previously validated in Iran, the Food Frequency Questionnaire (FFQ) (24) was utilized to collect the relevant diet-related data. Information about calorie intake, whole and refined grains, fruits and vegetables, simple sugars, fat, salty snacks, dairy products, and meats over the course of a year was assessed using these questionnaires. Portion sizes were adjusted to grams after accounting for household measurements. The number of calories and nutrients was evaluated using the US Department of Agriculture’s food composition table (FCT) (USDA, Release 11, 1994, modified for Iranian cuisine). For local goods that were not available in the FCT, the Iranian FCT was taken into consideration. Additionally, the Nutritionist 4 software (Axxya Systems, United States) was used to evaluate the intake of nutrients in children, and the intake of total selenium was examined.

### Statistical analysis

For the qualitative and quantitative variables, chi-square and independent *t*-test techniques were utilized to compare food intake and sociodemographic markers, respectively. The linear regression method was used to investigate the association between dietary intake of selenium and SA. Additionally, in order to look into how the FTO gene genotype affects the relationship between selenium intake and abortion, regression analysis was performed separately in individuals with AA/AT and TT genotypes according to the dominant genetic model. All analyses were performed using IBM SPSS version 27 at significant level of *p* < 0.05.

## Results

Table 1 displays the participants’ overall characteristics. The cases had a higher history of diabetes (31% vs. 23%, *p* = 0.001). There was no significant difference between the groups regarding age, weight, height, BMI, menstruation first age, Diastolic Blood Pressure (DBP), Systolic Blood Pressure (SBP), Hemoglobin (HGB), RBC: Red Blood Cell (RBC), hematocrit (HCT), White Blood Cell (WBC), Mean corpuscular volume (MCV), mean corpuscular hemoglobin (MCH), and mean corpuscular hemoglobin concentration (MCHC), platelet, lymphocyte, monocyte, Blood Urea Nitrogen (BUN), creatinine, TG (Triglycerides), cholesterol, Serum Glutamic Oxaloacetic Transaminase (SGOT), Serum Glutamate Pyruvate Transaminase (SGPT), Alkaline Phosphatase (ALP), High-Density Lipoprotein Cholesterol (HDLC), Low-density Lipoprotein cholesterol (LDL), PCR result, tobacco usage, hypertension, and intakes of carbohydrate, calorie, protein, fats, and selenium.

**Table 1:** General characteristics of the participants.

	Cases (*n* = 192)	Controls (*n* = 347)	*p*
Age (year)	35.84 ± 10.16	32.60 ± 12.86	0.07
Height (cm)	156.87 ± 6.19	156.80 ± 5.68	0.88
Weight (kg)	71.89 ± 10.51	70.35 ± 10.29	0.10
BMI (kg/m^2^)	29.16 ± 4.01	28.59 ± 3.96	0.11
Menstruation first age (year)	13.39 ± 1.51	13.27 ± 1.62	0.50
Right DBP1 (mmHg)	70.52 ± 9.33	70.34 ± 8.80	0.85
Right SBP1 (mmHg)	109.29 ± 14.03	108.75 ± 14.19	0.73
WBC (K/μL)	6.33 ± 1.52	6.15 ± 1.42	0.28
RBC (M/μL)	4.83 ± 0.40	4.85 ± 0.38	0.59
HGB (gr/dl)	13.35 ± 1.09	13.33 ± 0.96	0.28
HCT (%)	40.53 ± 2.88	40.55 ± 0.2.68	0.94
MCV (fl)	84.16 ± 6.04	83.75 ± 5.53	0.54
MCH (pg)	27.72 ± 2.42	27.55 ± 2.18	0.51
MCHC (gr)	32.91 ± 0.79	27.55 ± 2.18	0.62
Platelets (K/μL)	316.11 ± 66.48	308.20 ± 68.68	0.29
Lymphocyte (10^6^/L)	40.97 ± 8.07	41.23 ± 8.92	0.77
Monocyte (10^6^/L)	3.25 ± 1.03	3.28 ± 1.06	0.81
BUN (mg/dl)	12.50 ± 3.17	12.62 ± 3.51	0.74
Creatinine (mg/ml)	0.97 ± 0.11	0.96 ± 0.11	0.72
TG (mg/dl)	133.35 ± 79.82	121.55 ± 69.11	0.16
Cholesterol (mg/dl)	195.66 ± 37.24	194.52 ± 39.65	0.79
SGOT (IU/L)	19.04 ± 5.51	18.49 ± 4.87	0.35
SGPT (IU/L)	18.87 ± 7.62	17.70 ± 9.04	0.19
ALP (IU/L)	233.73 ± 76.95	220.18 ± 66.45	0.09
HDLC (mg/dl)	54.39 ± 10.78	55.76 ± 11.44	0.26
LDL (mg/dl)	114.59 ± 32.52	114.45 ± 33.39	0.97
PCR result (%) TT	94 (36.96)	40 (28.6)	0.33
PCR result (%) AA	22 (8.6)	14 (10.0)	
PCR result (%) AT	138 (54.1)	86 (61.4)	
Alcohol usage (yes, *n*, %)	17 (8.8)	28 (14.6)	0.11
Tobacco usage (yes, *n*, %)	20 (5.8)	8 (4.2)	0.27
Has Diabetes (yes, *n*, %)	81 (23.3)	60 (31.3)	0.03
Has Hypertension (yes, *n*, %)	89 (25.6)	52 (27.1)	0.39
Calorie (kcal/day)	2534.83 ± 437.91	2552.82 ± 674.69	0.74
Protein (g/day)	84.71 ± 21.34	83.79 ± 30.31	0.71
Carbohydrates (g/day)	359.44 ± 73.84	363.17 ± 99.81	0.66
Fats (g/day)	92.13 ± 21.74	94.16 ± 31.69	0.44
Selenium (mg/day)	96.22 ± 32.72	96.10 ± 38.43	0.97

Right SBP, Right-hand Systolic Blood Pressure; Right DBP, Right-hand Diastolic Blood Pressure; Hgb, Hemoglobin; WBC, White Blood Cell; RBC, Red Blood Cell; BUN, Blood Urea Nitrogen; TG, Triglyceride; HDL, High-density Lipoprotein cholesterol; LDL, Low-density Lipoprotein Cholesterol; SGOT, Serum Glutamic Oxaloacetic Transaminase; SGPT, Serum Glutamic Pyruvic Transaminase; ALP, Alkaline Phosphatase.

As shown in Table 2, no significant difference was observed in dietary intakes in carriers of AA/AT FTO genotypes. Dietary intake of selenium in the cases was partially lower than the controls in carriers of TT genotype of FTO rs9930609 polymorphism (90.3 ± 24.89 vs. 101.16 ± 36.6, *p* = 0.07).

**Table 2:** Dietary intake of the participants considering FTO rs9939609 genotypes.

	TT			AA			AT		
	Cases	Controls	*p*	Cases	Controls	*p*	Cases	Controls	*p*
Calorie (kcal/d)	2551.98 ± 406.06	2564.6 ± 592.36	0.89	2422.01 ± 286.18	2670.9 ± 821.83	0.24	2543.77 ± 451.05	2546.13 ± 701.83	0.97
Protein (g/day)	83.78 ± 17.17	84.17 ± 25.52	0.92	76.26 ± 14.58	88.87 ± 48.22	0.31	83.19 ± 19.53	83.85 ± 33.23	0.86
Carbohydrates (g/day)	361.24 ± 69.30	364.95 ± 100.38	0.82	344.24 ± 63.79	379.29 ± 122.09	0.31	363.18 ± 61.93	362.45 ± 89.15	0.95
Fats (g/day)	93.68 ± 16.39	95.28 ± 29.49	0.71	88.01 ± 11.89	97.49 ± 26.64	0.19	92.82 ± 26.98	93.10 ± 33.55	0.95
Selenium (mg/day)	90.3 ± 24.89	101.16 ± 36.6	0.07	91.59 ± 32.72	100.53 ± 38.15	0.49	101.43 ± 35.06	94.35 ± 38.69	0.19

Linear regression of the association between SA and selenium intake considering FTO genotypes for rs9939609 polymorphism is presented in Table 3. A negative association was found between SA and the intake of selenium in individuals with TT genotypes (*β* = −0.19, *p* = 0.04) (Model 1). The relationship remained significant after adjusting for age (Model 2), further adjustment for BMI and physical activity, smoking, and alcohol drinking (Model 3), and further adjustment for calorie intake (Model 4). There was no significant association between selenium intake and SA in individuals with AA and AT genotypes.

**Table 3:** Linear regression of the association between SA and selenium intake considering FTO genotypes for rs9939609 polymorphism.

	Model 1		Model 2		Model 3		Model 4	
	Beta	*p*	Beta	*p*	Beta	*p*	Beta	*p*
TT	−0.19	0.04	−0.19	0.04	−0.20	0.03	−0.21	0.04
AA	−0.07	0.69	−0.10	0.58	−0.12	0.54	0.05	0.83
AT	0.07	0.30	0.07	0.28	0.08	0.26	0.06	0.38

Model 1: Crude, Model 2: Further adjusted for age, Model 3: Further adjustment for BMI and physical activity, smoking, and alcohol drinking, Model 4: Further adjusted for calorie intake.

## Discussion

The aim of this study was the investigate the associations between FTO genotypes, selenium, and SA. The result indicated that there was a negative correlation between SA and selenium intake in people with TT genotypes. There was no significant association between selenium intake and SA in individuals with AA and AT genotypes.

The effects of diet components on the risk of SA have been widely reported (13, 25). The results of this study on the association between selenium and SA are in line with those of a number of other studies (26, 27). Similarly, some previous studies have suggested that the association between SA risk and dietary components may be influenced by FTO genotype (28, 29).

Selenium was reported to be involved in pregnancy outcomes (30–33). Selenium is essential for the immune system’s reaction and infection resistance (34). The link between selenium deficiency and SA has been explained by at least three different processes. The first is the deterioration of selenium-dependent antioxidant capacity, which damages DNA and cell membranes (30). The second is a reduction in antithrombin III activity as a result of selenium deficiency (35) and the third is the decreased activity of selenium-containing enzymes, or selenioenzymes, which have been shown to suppress the expression of pro-inflammatory genes linked to unfavorable pregnancy outcomes (34, 36, 37). However, the FTO gene was identified as a separate genetic risk factor for obesity for the first time in a genome-wide association study (38). More recently, polymorphisms in the FTO gene have been found to be associated with a variety of diseases, including cancer, diabetes mellitus, heart attack, and kidney failure. However, the exact function of the FTO gene in biology is still unknown (39). The process of nucleic acid demethylation involves the FTO gene. The FTO gene is a possible candidate gene for SA susceptibility since proper fetal development is dependent on DNA methylation. Furthermore, FTO’s possible involvement in fetal development is further supported by the high expression of the protein in human placentas and its correlation with larger fetal weights and lengths (40). Understanding the correlation between SA and dietary selenium intake in people with different genotypes will help us to plan a tailored diet to reduce the incidence of SA in at-risk populations.

Regarding the possible mechanisms, selenium has been identified to play several critical roles in maintaining pregnancy, including antioxidant defense, immune regulation, and thyroid hormone metabolism. Selenium deficiency may be associated with reduced activity of glutathione peroxidases, leading to increased oxidative stress factor that might lead to placental damage. Altered immune responses and impaired thyroid function may be associated with low selenium levels, which can be the reason for implantation not taking place or it might lead to an imbalance in hormonal levels, which is one of the causes of SA (41).

**Figure 2:**
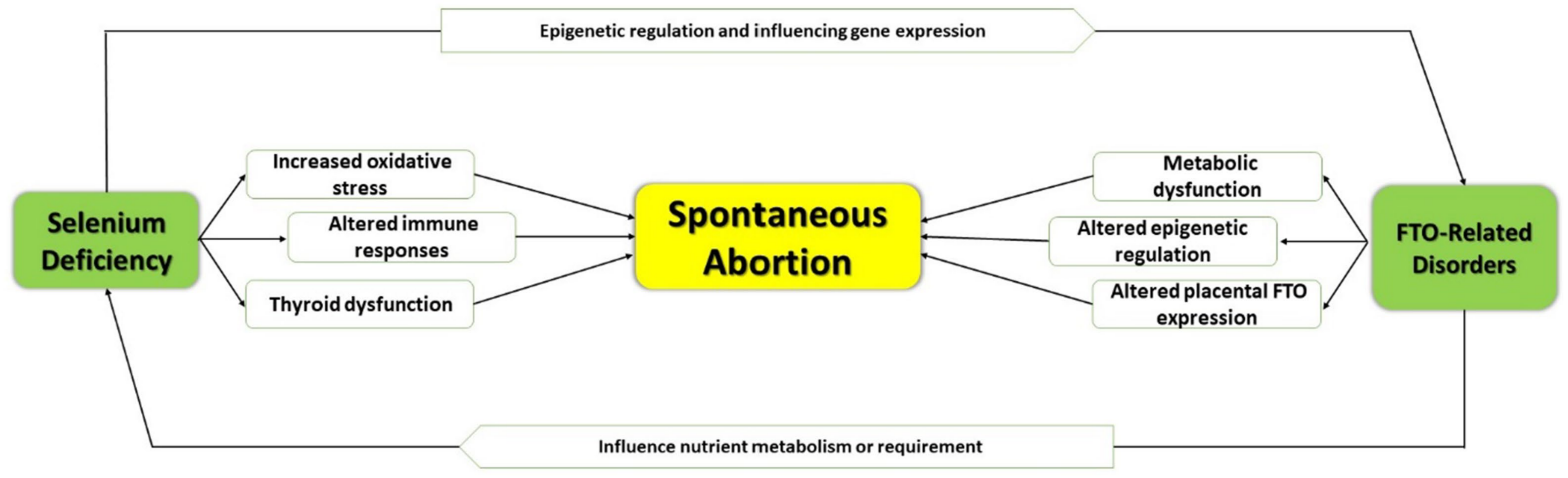
Epigenetic regulation and influencing expression.

Though the FTO gene is mainly identified with obesity and the regulation of metabolism, pregnancy outcomes can be influenced indirectly (23). Variants of the FTO gene are associated with higher BMI and metabolic disturbances, factors that increase the risk of complications in pregnancy. In addition, FTO as an RNA demethylase could affect the expression of genes involved in placental function and fetal development through epigenetic mechanisms leading to an increased risk of spontaneous abortion. Yet, another interaction between selenium and FTO may be genetic variations that determine the metabolism or utilization of selenium, hence pregnancy outcomes. Despite these seemingly reasonable biological pathways, the evidence has remained limited; therefore, further research into how selenium and FTO interact with the risk of SA may be necessary (19, 41, 42) (Figure 2).

To the best of our knowledge, this is the first study to investigate how the rs9939609 polymorphism in the FTO gene affects the relationship between selenium and SA. A large number of participants and adjustment for potential confounders were other strengths of this study. However, the FFQ used to assess diet is a self-report tool and is subject to some errors such as recall bias and under-reporting of dietary intake in obese and overweight participants. Furthermore, the selection of individuals from a single hospital may make it difficult to generalize the findings. There is a need to conduct more long-term research to validate these results.

## Conclusion

According to the study, individuals with TT genotypes showed a negative link between SA and selenium intake, whereas no significant association was observed between SA and selenium intake in individuals with AA and AT genotypes. These findings emphasize the importance of personalized diets in preventing SA. If the results are confirmed in future studies, we can use selenium-rich diets and selenium supplements to prevent SA in people who have a genetic predisposition. However, more research is needed to completely comprehend the connection between dietary factors and FTO gene polymorphisms in determining the risk of SA.

## Data Availability

The original contributions presented in the study are included in the article/supplementary material, further inquiries can be directed to the corresponding author.

## References

[1] Carranza-Lira S Blanquet J Tserotas K Calzada L. Endometrial progesterone and estradiol receptors in patients with recurrent early pregnancy loss of unknown etiology--preliminary report. Med Sci Monit. (2000) 6:759–62. PMID: 11208405

[2] Ugurlu EN Ozaksit G Karaer A Zulfikaroglu E Atalay A Ugur M. The value of vascular endothelial growth factor, pregnancy-associated plasma protein-a, and progesterone for early differentiation of ectopic pregnancies, normal intrauterine pregnancies, and spontaneous miscarriages. Fertil Steril. (2009) 91:1657–61. doi: 10.1016/j.fertnstert.2008.02.002, PMID: 18402945

[3] Practice Committee of the American Society for Reproductive Medicine. Evaluation and treatment of recurrent pregnancy loss: a committee opinion. Fertil Steril. (2012) 98:1103–11. doi: 10.1016/j.fertnstert.2012.06.048 22835448

[4] The ESHRE Guideline Group on RPL Bender Atik R Christiansen OB Elson J Kolte AM Lewis S. ESHRE guideline: recurrent pregnancy loss. Hum Reprod Open. (2018) 2018:hoy004. doi: 10.1093/hropen/hoy004, PMID: 31486805 PMC6276652

[5] Quenby S Gallos ID Dhillon-Smith RK Podesek M Stephenson MD Fisher J. Miscarriage matters: the epidemiological, physical, psychological, and economic costs of early pregnancy loss. Lancet. (2021) 397:1658–67. doi: 10.1016/S0140-6736(21)00682-6 33915094

[6] Alipanahpour S Tayebi N Zarshenas M Akbarzadeh M. Short-term physical and psychological health consequences of induced and spontaneous abortion: a cross-sectional study. Shiraz E-Med J. (2021) 22:e111809. doi: 10.5812/semj.111809

[7] Wang Q Liu F Tuo Y Ma L Feng X. Associations between obesity, smoking behaviors, reproductive traits and spontaneous abortion: a univariable and multivariable Mendelian randomization study. Front Endocrinol. (2023) 14:1193995. doi: 10.3389/fendo.2023.1193995 PMC10400331 37547316

[8] Wang JX Davies MJ Norman RJ. Obesity increases the risk of spontaneous abortion during infertility treatment. Obes Res. (2002) 10:551–4. doi: 10.1038/oby.2002.74 12055331

[9] Metwally M Ong KJ Ledger WL Li TC. Does high body mass index increase the risk of miscarriage after spontaneous and assisted conception? A meta-analysis of the evidence. Fertil Steril. (2008) 90:714–26. doi: 10.1016/j.fertnstert.2007.07.1290, PMID: 18068166

[10] Turner MJ Fattah C O’Connor N Farah N Kennelly M Stuart B. Body mass index and spontaneous miscarriage. Eur J Obstet Gynecol Reprod Biol. (2010) 151:168–70. doi: 10.1016/j.ejogrb.2010.04.021 20488611

[11] de La Rochebrochard E Thonneau P. Paternal age and maternal age are risk factors for miscarriage; results of a multicentre European study. Hum Reprod. (2002) 17:1649–56. doi: 10.1093/humrep/17.6.1649 12042293

[12] Mosayebi-Molasaraie M Doosti-Irani A Pilevari S Cheraghi Z. Predictors of miscarriage in the west of Iran: a case-control study. J Iran Med Council. (2023) 6:362–8. doi: 10.18502/jimc.v6i2.12247

[13] Pieczyńska J Grajeta H. The role of selenium in human conception and pregnancy. J Trace Elem Med Biol. (2015) 29:31–8. doi: 10.1016/j.jtemb.2014.07.003, PMID: 25175508

[14] Mojadadi A Au A Salah W Witting P Ahmad G. Role for selenium in metabolic homeostasis and human reproduction. Nutrients. (2021) 13:3256. doi: 10.3390/nu13093256, PMID: 34579133 PMC8469766

[15] Dahlen CR Reynolds LP Caton JS. Selenium supplementation and pregnancy outcomes. Front Nutr. (2022) 9:1011850. doi: 10.3389/fnut.2022.1011850, PMID: 36386927 PMC9659920

[16] Atarod Z Emadi N Saeedi Saravi SS Modanlookordi M Shokrzadeh M. Copper and selenium levels in women with second-trimester induced abortion in Mazandaran, 2009: a case control study. Pharmaceut Biomed Res. (2015) 1:44–7. doi: 10.18869/acadpub.pbr.1.1.44

[17] Andraweera PH Dekker GA Jayasekara RW Dissanayake VH Roberts CT. The obesity-related FTO gene variant associates with the risk of recurrent miscarriage. Acta Obstet Gynecol Scand. (2015) 94:722–6. doi: 10.1111/aogs.12640 25845303

[18] Liu C Mou S Pan C. The FTO gene rs9939609 polymorphism predicts risk of cardiovascular disease: a systematic review and meta-analysis. PLoS One. (2013) 8:e71901. doi: 10.1371/journal.pone.0071901, PMID: 23977173 PMC3747067

[19] Qiu W Zhou Y Wu H Lv X Yang L Ren Z. RNA demethylase FTO mediated RNA m6A modification is involved in maintaining maternal-fetal interface in spontaneous abortion. Front Cell Dev Biol. (2021) 9:617172. doi: 10.3389/fcell.2021.617172, PMID: 34350169 PMC8326377

[20] Czajkowski P Adamska-Patruno E Bauer W Fiedorczuk J Krasowska U Moroz M. The impact of FTO genetic variants on obesity and its metabolic consequences is dependent on daily macronutrient intake. Nutrients. (2020) 12:3255. doi: 10.3390/nu12113255, PMID: 33114268 PMC7690875

[21] Sullivan KM Dean A Soe MM. On academics: OpenEpi: a web-based epidemiologic and statistical calculator for public health. Public Health Rep. (2009) 124:471–4. doi: 10.1177/003335490912400320, PMID: 19445426 PMC2663701

[22] Di Cintio E Parazzini F Chatenoud L Surace M Benzi G Zanconato G. Dietary factors and risk of spontaneous abortion. Eur J Obstet Gynecol Reprod Biol. (2001) 95:132–6. doi: 10.1016/S0301-2115(00)00363-8 11267735

[23] Azizi-Tabesh G Sadeghi H Farhadi A Heidari MF Safari A Shakouri Khomartash M. The obesity associated FTO gene polymorphism and the risk of preeclampsia in Iranian women: a case-control study. Hypertens Pregnancy. (2023) 42:2210685. doi: 10.1080/10641955.2023.2210685, PMID: 37160708

[24] Shahar D Shai I Vardi H Brener-Azrad A Fraser D. Development of a semi-quantitative food frequency questionnaire (FFQ) to assess dietary intake of multiethnic populations. Eur J Epidemiol. (2003) 18:855–61. doi: 10.1023/A:1025634020718 14561044

[25] Gupta S Agarwal A Banerjee J Alvarez JG. The role of oxidative stress in spontaneous abortion and recurrent pregnancy loss: a systematic review. Obstet Gynecol Surv. (2007) 62:335–47. doi: 10.1097/01.ogx.0000261644.89300.df 17425812

[26] Abdulah R Noerjasin H Septiani L Mutakin DIR Suradji EW Puspitasari IM. Reduced serum selenium concentration in miscarriage incidence of Indonesian subjects. Biol Trace Elem Res. (2013) 154:1–6. doi: 10.1007/s12011-013-9701-0 23695728

[27] Güvenç M Güven H Karataş F Aygün AD Bektaş S. Low levels of selenium in miscarriage. J Trace Elements Exp Med. (2002) 15:97–101. doi: 10.1002/jtra.10004

[28] Andraweera PH Dekker GA Leemaqz S McCowan L Roberts CT SCOPE Consortium. The obesity associated FTO gene variant and the risk of adverse pregnancy outcomes: evidence from the SCOPE study. Obesity. (2016) 24:2600–7. doi: 10.1002/oby.21662, PMID: 27768255

[29] Hubacek JA Kasparova D Dlouha D Pikhart H Bobak M Fait T. FTO gene variant and risk of spontaneous abortion. Acta Obstet Gynecol Scand. (2016) 95:118. doi: 10.1111/aogs.12777, PMID: 26399564

[30] Barrington J Lindsay P James D Smith S Roberts A. Selenium deficiency and miscarriage: a possible link? BJOG Int J Obstet Gynaecol. (1996) 103:130–2. doi: 10.1111/j.1471-0528.1996.tb09663.x, PMID: 8616128

[31] Negro R Greco G Mangieri T Pezzarossa A Dazzi D Hassan H. The influence of selenium supplementation on postpartum thyroid status in pregnant women with thyroid peroxidase autoantibodies. J Clin Endocrinol Metabol. (2007) 92:1263–8. doi: 10.1210/jc.2006-1821 17284630

[32] Rayman MP Bode P Redman CW. Low selenium status is associated with the occurrence of the pregnancy disease preeclampsia in women from the United Kingdom. Am J Obstet Gynecol. (2003) 189:1343–9. doi: 10.1067/S0002-9378(03)00723-3, PMID: 14634566

[33] Tara F Rayman M Boskabadi H Ghayour-Mobarhan M Sahebkar A Yazarlu O. Selenium supplementation and premature (pre-labour) rupture of membranes: a randomised double-blind placebo-controlled trial. J Obstet Gynaecol. (2010) 30:30–4. doi: 10.3109/01443610903267507, PMID: 20121500

[34] Rayman MP. Food-chain selenium and human health: emphasis on intake. Br J Nutr. (2008) 100:254–68. doi: 10.1017/S0007114508939830, PMID: 18346308

[35] Aursnes I Smith P Arnesen H Åkesson B. Correlation between plasma levels of selenium and antithrombin-III. Eur J Haematol. (1988) 40:7–11. doi: 10.1111/j.1600-0609.1988.tb00789.x, PMID: 3342861

[36] Moses EK Johnson MP Tømmerdal L Forsmo S Curran JE Abraham LJ. Genetic association of preeclampsia to the inflammatory response gene SEPS1. Am J Obstet Gynecol. (2008) 198:336.e1. e5–5. doi: 10.1016/j.ajog.2007.09.024 18068137

[37] Vunta H Davis F Palempalli UD Bhat D Arner RJ Thompson JT. The anti-inflammatory effects of selenium are mediated through 15-deoxy-Δ12, 14-prostaglandin J2 in macrophages. J Biol Chem. (2007) 282:17964–73. doi: 10.1074/jbc.M703075200, PMID: 17439952

[38] Frayling TM Timpson NJ Weedon MN Zeggini E Freathy RM Lindgren CM. A common variant in the FTO gene is associated with body mass index and predisposes to childhood and adult obesity. Science. (2007) 316:889–94. doi: 10.1126/science.1141634, PMID: 17434869 PMC2646098

[39] Shen L Song C-X He C Zhang Y. Mechanism and function of oxidative reversal of DNA and RNA methylation. Annu Rev Biochem. (2014) 83:585–614. doi: 10.1146/annurev-biochem-060713-035513, PMID: 24905787 PMC4786441

[40] Bassols J Prats-Puig A Vázquez-Ruíz M García-González M Martínez-Pascual M Avellí P. Placental FTO expression relates to fetal growth. Int J Obes. (2010) 34:1365–70. doi: 10.1038/ijo.2010.62, PMID: 20351740

[41] Favara G Maugeri A Magnano San Lio R Barchitta M Agodi A. Exploring gene–diet interactions for mother–child health: a systematic review of epidemiological studies. Nutrients. (2024) 16:994. doi: 10.3390/nu16070994 38613027 PMC11013682

[42] Franzago M Fraticelli F Marchioni M Di Nicola M Di Sebastiano F Liberati M. Fat mass and obesity-associated (FTO) gene epigenetic modifications in gestational diabetes: new insights and possible pathophysiological connections. Acta Diabetol. (2021) 58:997–1007. doi: 10.1007/s00592-020-01668-5, PMID: 33743080 PMC8272710

